# Turning the Mirror on the Architects: A Study of the Open-Plan Office and Work Behaviors at an Architectural Company

**DOI:** 10.3389/fpsyg.2018.02178

**Published:** 2018-11-20

**Authors:** Dorota Węziak-Białowolska, Zhao Dong, Eileen McNeely

**Affiliations:** ^1^Sustainability and Health Initiative (SHINE), Department of Environmental Health, Harvard T. H. Chan School of Public Health, Harvard University, Boston, MA, United States; ^2^Department of Environmental Health, Harvard T. H. Chan School of Public Health, Harvard University, Boston, MA, United States

**Keywords:** open-plan office, privacy, office density, affective events theory, irritability, fit into workspace, work performance, social relations at work

## Abstract

Following the rising cost of real estate and a desire to increase collaboration and communication among employees, the open-plan office has been trending over the past decades. Research about the impact of the open-plan office on humans is equivocal in endorsing this trend. The mixed results are further confounded following the specific job requirements, such as the need for privacy in jobs requiring a high level of concentration or, in contrast, the need for open workspace in jobs benefitting from team work and knowledge sharing. This study aims to understand the relationship between perceptions of three characteristics of the open-plan office (acoustical privacy, visual privacy, and office density), and the impact they yield on employees' judgment as well as affect-driven behaviors. The study benefits from the data from 456 employees located in 20 regional office locations within the same architectural firm. The restriction to employees of a design firm enables examinations of participants, who are already sensitive to the impacts of space by the nature of their work. The variables of interest included employee perception of the workspace (privacy, office density, and fit into workspace), employee rating of social relationships, self-reported mood (irritability) and optimal functioning (number of limited ability days), and work impacts (job satisfaction, work engagement, and job performance). The Model of behavior in an open-plan office setting based on affective events theory is adopted. Mediation roles of irritability and perception of fit into the workspace are examined. Structural equation modeling is applied to test the joint significance of the association between independent and dependent variables (direct effect) and the association between independent variables, mediator, and dependent variables (indirect effect). Nested structure of the data is accounted for by adjusting the standard errors for clustering. The significance of indirect and total effects is evaluated by the bootstrapping method. Our results show that working in the open-plan office limits the experience of privacy and intensifies the perception of intrusion among employees of an architectural company, mostly architects and designers. Additionally, employees' perception of lack of privacy and high office density negatively affect job satisfaction, work engagement, and internal work relation as well as increases the number of limited ability days. Interestingly, the lack of privacy and high office density seem to positively affect expressive personal relations among coworkers and job performance. We find supporting evidence for mediation roles of negative emotions, that is, irritability and perception of fit into the workspace.

## Introduction

From the perspective of real estate costs and environmental footprint, the open-plan office has been widely promoted. However, research findings on its impact on business, health, and social relationships outcomes have been mixed. On the one hand, some studies show that employees, who are satisfied and content with their open-plan office, reported higher job satisfaction (Veitch et al., 2007; Bangwal et al., 2017), increased organizational commitment (Bangwal et al., 2017), positive coworkers' relations and collaboration (Zahn, 1991), and better rapport with supervisors (Sundstrom et al., 1980), mainly because of close proximity to other coworkers. On the other hand, a bunch of studies indicate that enclosed private offices outperform open-plan offices with respect to health status (Bodin Danielsson and Bodin, 2008), sick leave rates (Bodin Danielsson et al., 2014), job satisfaction (Oldham and Brass, 1979; De Croon et al., 2005; Bodin Danielsson and Bodin, 2008), and social relations (Oldham and Brass, 1979; Sundstrom et al., 1982; Kaarlela-Tuomaala et al., 2009), mainly due to lack of privacy in open-plan office environment (Rashid and Zimring, 2008; Laurence et al., 2013).

Since results that have been reported are likely to be influenced by the specificity of an organization, the generalization of results is needed. This could be achieved by conducting a systematic literature review, meta-analysis, or one company multisite study. While the systematic literature review or meta-analysis summarizes studies already conducted (Borenstein et al., 2009), the one company multisite study enables examination of employees sharing similar job characteristics—resulting from the purpose of the business—but performing tasks in multiple locations, thus facilitating formulation of more universal conclusions.

In this study, by looking at data from 456 employees located in 20 regional office locations within the same architectural firm, we applied the third approach. We aimed to examine the impact of working in an open-plan office at an architectural company, that is, among those who are responsible for designing office spaces. As designers, these employees would presumably be more perceptible to their surroundings, providing a unique opportunity for new insights into space. Further, given that the requirements of the job would be similar, such as the need for teamwork, these homogeneous conditions would avoid the limitations of other studies that included multiple jobs with different space needs. This study offered a unique opportunity to learn about open-plan office design among similar participants with similar jobs and among people likely to be sensitive to environmental cues.

## Background

### Concerns for privacy and spatial density in open-plan offices

There is equivocal evidence on the impacts of the open-plan office on humans. In the pursuit of explanations of effects of the open-plan office on employees, scholars distinguished several dimensions through which physical work environment (open-plan office in particular) may affect employees' performance and behaviors. Among them, the most-often mentioned are privacy at work, crowding, and office density (Ashkanasy et al., 2014; Khazanchi et al., 2018).

There is a common agreement that physical work environment affects privacy at work (Ashkanasy et al., 2014; Bodin Danielsson et al., 2015; Khazanchi et al., 2018). Privacy is defined in several ways. It is described as a condition of physical, psychological, and informational separation of a person from others and of others from a person (Newell, 1994). It is also described *as “the selective control of access to the self, involving dialectic, optimization, and multimodal processes*,” which serves three functions: (1) management of social interaction, (2) establishment of plans and strategies for interacting with others, and (3) development and maintenance of self-identity (Altman, 1977, 67–68). Sundstrom et al. (1980) distinguish psychological privacy and architectural privacy. The former is related to Altman's (1977) sense of control of access and the latter refers to physical isolation from people, defined as absence of external acoustical and visual stimuli.

Another characteristic of an employee's physical work environment is spatial density. Spatial density is defined in terms of usable office space (Oldham, 1988). Reduced space implies increased density but also further likely increased potential for crowding, distraction (Khazanchi et al., 2018), and conflict (Ayoko et al., 2014).

Ashkanasy et al. (2014) argued that characteristics of the physical work environment, in particular, high-density open-plan offices, may impact employees' attitudes, behaviors, performances, and well-being. To examine impacts, the researchers suggested an approach based on the affective events theory of Weiss and Cropanzano (1996). Starting with an assertion that an open-plan office is usually associated with lack of privacy and proximity to other employees, they argued that consequently it induces distractions, interruptions, and invasions of employees' personal space in the office. They claimed further that not only can these circumstances lead directly to the formation of employee attitude over time (which they called the perception of fit into workspace) and to certain behaviors (e.g., conflict, withdrawal, and territorial behaviors), but may also result in negative affective reactions, such as anger and frustration. Those negative reactions might subsequently translate into negative employee attitudes (e.g., perception of decreased fit into workspace) and finally lead to the so-called, by Ashkanasy et al. (2014), judgment-driven behaviors.

Based on the arguments presented previously, the following were hypothesized:

H1. Employees experiencing lack of architectural privacy, that is, working in an open-plan office, compared with those working in private offices will report more often of the following:

H1.1. Issues with privacy and office density;
H1.2. Negative moods;
H1.3. Difficulties with concentration;
H1.4. Lower perception of attractiveness of workspaces;
*H1.5. Worse performance outcomes*.

While Ashkanasy et al. (2014) focus on negative affective consequences of working in open-plan office, Veitch (2018) indicates positive affective outcomes. The perceptions of attractiveness of workspaces, as named by Veitch (2018), can lead to improved mood, which, in turn, can lead to environmental satisfaction and further greater job satisfaction, improved work engagement, and reduced intent to quit. As Veitch's (2018) concept of attractiveness of workspace seems to be conceptually similar to the perception of fit into workspace in the Ashkanasy et al.'s (2014), we formulated an additional hypothesis:

*H2. Employees reporting positive attitudes toward attractiveness of workspace report better performance outcomes*.

### Social bonds instead of walls

There is some confusion about what happens to social relationships when the walls come down in open-plan offices. Two approaches, as proposed by Oldham and Brass (1979), have been used: social relations approach and sociotechnical approach. The former focuses on a positive effect that the lack of walls has on social relations and interactions between employees including supervisor-employee feedback. It is further believed that the open-plan office increases motivation, performance, and job satisfaction. The latter, that is, sociotechnical approach, associates lack of privacy with a high level of interference, leading to decreased autonomy, supervisor's and coworker's feedback, job satisfaction, lower motivation, and worse job performance.

Although there is an argument that open-plan offices should eliminate status barriers, facilitate communication, and encourage collaborative work (Veitch, 2018), mixed findings have been reported. Some studies find that a positive impact of facilitated interactions in open-plan office is easily offset by negative impact of noise and limited privacy (Kim and de Dear, 2013). There are also studies showing limited evidence of detrimental effect of working in open-plan offices with social relationships (Sundstrom et al., 1980; De Croon et al., 2005) and inconsistent or non-significant evidence on the effect of office layout on communication, autonomy, stress due to crowding, performance, and health (Duval-Early and Benedict, 1992; Leather et al., 2003; De Croon et al., 2005).

These results are in line with the theoretical assertion of Khazanchi et al. (2018) that the channel through which physical office space affects work relationships in terms of communication, territoriality, and self-regulatory resources can be twofold: (1) relationship-building and (2) relationship-straining. However, Khazanchi et al. (2018) add that while examining the impact of spatial design of open-plan office on work relationships, two types of relationships should be distinguished: expressive and instrumental working relations. The former relate to sharing nonwork-related communication, a sense of personal identification, and emotional attachment, for example, friendship. The latter are conceptualized as work-related interdependence and task-related information sharing.

As Khazanchi et al.'s (2018) model has not been empirically validated, in this paper we aim to test it with respect to two types of relationships distinguished–expressive and instrumental working relations. We hypothesize,

*H3. Close proximity to other employees (i.e., lack of privacy and increased office density) increases the likelihood of voluntary (expressive) ties with coworkers and support received from supervisors (instrumental ties)*.

## Conceptual research model

In the present study, we aimed to investigate on how the experience of limited privacy at work and office density (associated with working in the open-plan office) is related to job satisfaction, work engagement, job performance, self-reported limited ability days, and social relations outcomes among employees of a global architecture company. In particular, our intention was to examine reactions of employees, mostly architects, to working in an open-plan office. To this aim, we applied the model of behavior in open-plan office settings based on the affective events theory, originally proposed by Weiss and Cropanzano (1996) and adopted to the open-plan office concept by Ashkanasy et al. (2014, 1178). Weiss and Cropanzano's model focuses on the bearing of particular work-related situations or events on employees' emotions and moods at work that further translate into their behaviors. Despite highlighting “affective events” in the name of the model, Ashkanasy et al. (2014) explain that not only specific events, but also workplace circumstances and features, such as high-density open-plan offices, noise, and lack of privacy, may be perceived as triggers for negative emotions. The negative emotions, suggested by Ashkanasy et al. (2014), were anger and frustration. However, we used irritability, which is a prolonged, negative, emotional state or ability to react to changes in the environment (Kuczynski and Kolakowsky-Hayner, 2011). Since irritability refers directly to the reactions to the environment, it seemed more appropriate for this study.

**Figure 1:**
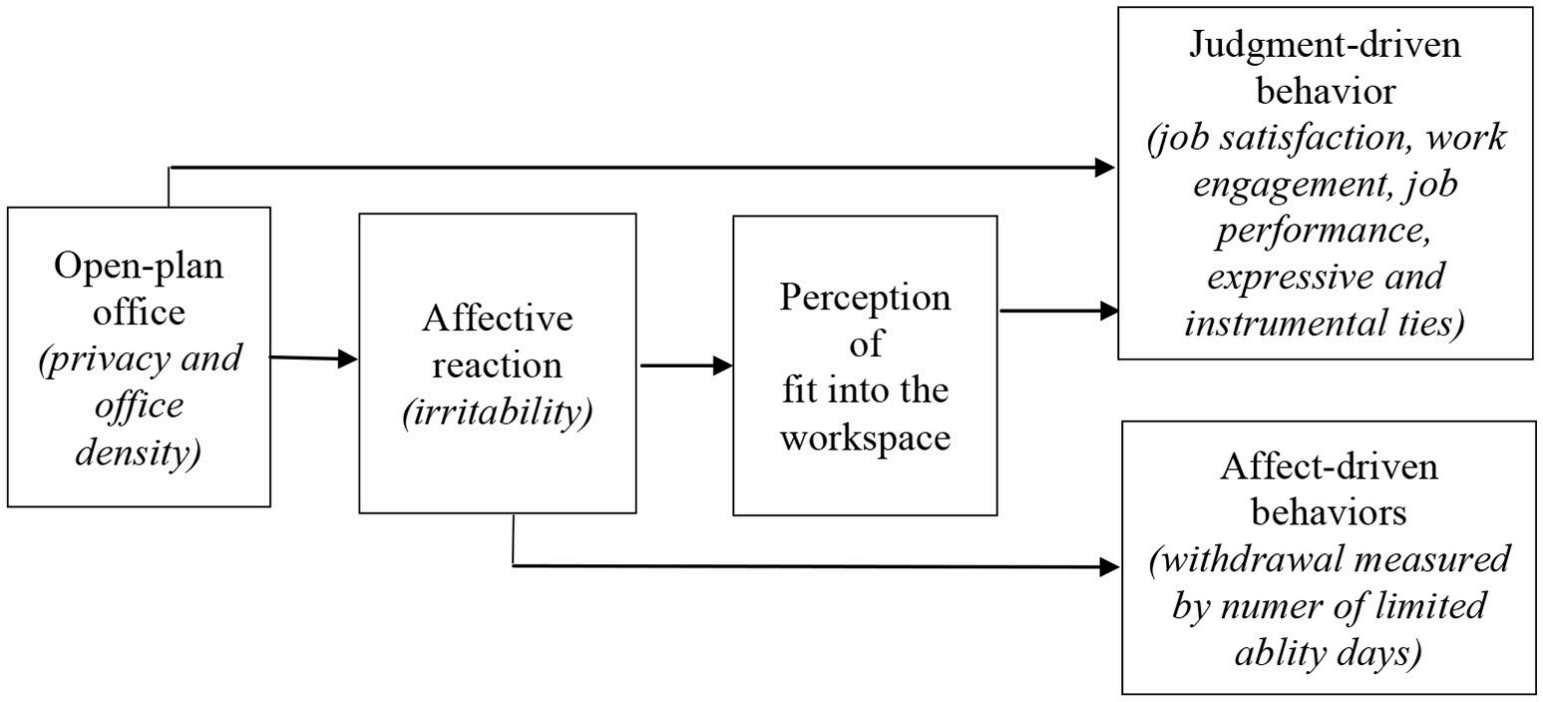
Conceptual research model of the effect of open-plan office on behaviors in the workplace - based on Ashkanasy et al. (2014), 1178.

Following Ashkanasy et al.'s (2014) model, the characteristics of open-plan offices can directly lead to the formation of employee attitude. It is referred to as perception of fit into the workspace and also, as noted by Veitch (2018, 83), (if satisfied) it may contribute to greater job satisfaction, increased affective organizational commitment, and reduced intent to turnover. According to Ashkanasy et al. (2014), features of open-plan offices can affect employees' behaviors directly and indirectly through negative emotions and perception of fit into the workspace. Therefore, two types of behaviors are distinguished: (1) judgment-driven behaviors (e.g., performance) and (2) affect-driven behaviors (e.g., withdrawal). While judgment-driven behaviors are affected by both emotions and perception of fit, the affect-driven behaviors are affected by emotions only (Figure 1).

Since Ashkanasy et al.'s (2014) model does not include components of work relationships, we capitalized on some elements of the spatial model of work relationships of Khazanchi et al. (2018). This model attempts to conceptualize the impact of open-plan office on work relationships. In particular, it focuses on the trade-offs associated with dimensions of the open-plan office and the relational costs and benefits associated with them.

Based on the model, we added positive work relationships to the judgment-driven behaviors. Following Khazanchi et al. (2018), we distinguished between expressive and instrumental ties. While the former relate to nonwork-related communication, the latter refer to work-related ties.

Based on the research model presented in Figure 1, we formulated additional hypotheses concerning the processes (indicated by arrows in Figure 1) through which open-plan office employees may be affected by two dimensions of this workspace (privacy and office density). We hypothesized that:

*H4. Lack of privacy and excessive office density will contribute to increased irritability*.
*H5. Increased irritability will reduce perception of fit into the workspace*.
*H6. Increased irritability will negatively impact affect-driven behaviors*.
*H7. Lack of privacy and excessive office density will directly and indirectly negatively contribute to judgment-driven behaviors (job satisfaction, work engagement, job performance, and expressive and instrumental ties)*.
*H8. Lack of privacy and excessive office density will directly and indirectly contribute negatively to affect-driven behavior (withdrawal measured by number of limited ability days)*.

Hypotheses H7 and H8 assume that irritability and perception of fit into the workspace (only in the case of H7) will mediate the relationships between lack of privacy and excessive office density with specific behaviors.

## Material and methods

### Participants

We surveyed employees at one of the top 30 US architecture companies that provides high-performance building design, research, and consulting services in the United States and Europe. The mission of the company comprises elements, such as thriving in a collaborative environment, fostering an open and collaborative culture, collaborative innovation through diversity, passion for creativity, addressing complex challenges creatively and collectively, and making a positive impact on the world through design. All these elements highlight the importance of collaboration, supportive culture, and creative environment for achieving the company's goals and employee well-being. In line with this, 86.7% of employees declared working in the open-plan office.

Each employee received an e-mail with invitation to participate in the survey. Only those who agreed were asked about working conditions, job resources, health, well-being, and socioeconomic characteristics. In total, 456 employees located in 20 regional offices participated in the survey in 2016 (average number of persons working in one location was 21.6, median was 31, minimum was 1, and maximum was 85 persons). Six employees, who worked mainly from home, were excluded from the analysis. The raw data supporting the conclusions of this manuscript will be made available by the corresponding author, without undue reservation, to any qualified researcher.

Informed written consent was obtained from the participants. Our study protocol was reviewed and approved by the Harvard Longwood Medical Area Institutional Review Board. Table 1 presents descriptive statistics of the participants.

**Table 1:** Descriptive statistics of participants.

**Variable/Levels**	**%**
**Gender**	
Female	39.4
Male	59.0
Deferred	1.6
**Race**	
White	72.8
Asian	6.1
Black	5.5
Other	15.6
**Education**	
High school or equivalent	1.2
Some college	6.1
Associate degree	6.5
Bachelor's degree	48.6
Graduate school education	37.6
**Marital status**	
Married	62.7
Widowed	0.9
Divorced	5.2
Separated	1.7
Never Married	20.5
Non-married partner	9.0
**Department**	
Architecture	49.3
Energy/Software	12.5
Engineering	15.3
Other	22.9
**Job title**	
Architect/Designer	60.2
Engineer	15.3
Project Director/Leader	20.4
Other	4.1
**Having job that requires being creative**	86.8
**Working in an open-plan office**	86.8

### Measures

#### Open-plan office

Open-plan office was assessed by a question regarding the design of the participant's current primary workspace. Respondents were asked to choose one or more options among the following: (1) an enclosed single-person office (assigned only to me), (2) an enclosed office shared with others, (3) a permanently assigned workstation or cubicle (assigned only to me), (4) a space permanently assigned to me and others, (5) an unassigned space, (6) I have a home office, where I work full time, and (7) other. Options presented to respondents have been consulted with the facility manager and the human resource manager and correspond to the common way of describing workspaces in the examined organization. Responses (3), (4), and (5) were indicative of working in an open-plan office. Responses (1) and (2) were indicative of working in a private office, either shared or single-person, henceforth, called a private office.

About 86.8% employees work in an open-plan office, with from 66.7% up to 100% of employees working in an open-plan office at different localizations (in one localization with none of five persons working in an open-plan office).

#### Dimensions of the physical work environment

Three complementary indicators were considered: visual privacy, acoustical privacy, and office density. Respondents were asked to assess how much each of these physical characteristics of workspace interfered with their ability to perform their duties (scale from 1 = not at all to 10 = to a great extent). These three indicators were examined separately in order to unravel their effects on behaviors. Another reason for such an approach was a considerable correlation among them (Pearson's correlation coefficients: 0.54 for acoustical privacy and office density, 0.73 for visual density and office density, and 0.78 for visual privacy and acoustical privacy), which might influence negatively the estimates in the path model used (i.e., issue of multicollinearity).

#### Affective reaction

A single question on the frequency of irritability experienced over the past 30 days at work was used (during the past 30 days, how much have you experienced irritability while at work on a scale of 1–7, where 1 = not at all to 7 = frequently).

#### Perception of fit into the workspace

Perception of fit into the workspace was measured using four questions: (1) *Overall, the physical space I work in enhances my individual work effectiveness*, (2) *Overall, the physical space where I work is an attractive aspect of my job*, (3) *The physical space where I work embodies the values of the organization I work for*, and (4) *Overall, I am satisfied with my work space*. These questions were measured on a 10-point scale, where 1 = extremely dissatisfied and 10 = extremely satisfied.

Personal fit scale to the workspace was tested using confirmatory factor analysis (CFA) and showed sufficient psychometric properties in our study (Comparative Fit Index (CFI) = 0.995, Tucker-Lewis Index (TLI) = 0.986, Root Mean Square Error of Approximation (RMSEA) = 0.096, Standardized Root Mean Square Residual (SRMR) = 0.009, Cronbach's alpha = 0.942).

#### Outcomes—judgment-driven behaviors

Five judgment-driven behaviors were examined: job satisfaction, work engagement, job performance, expressive ties, and instrumental ties.

Job satisfaction was assessed by a single question: *All in all, how satisfied are you with your job*? Response options ranged from 0 = not at all satisfied to 10 = extremely satisfied.

Work engagement was measured using the nine-item Utrecht Work Engagement Scale (Schaufeli, 2006), with higher values indicating higher work engagement. Exemplary statements assessed by employees comprise: *At my work, I feel bursting with energy; My job inspires me* (0 = I do not feel this at all; 6 = I feel this all the time). The work Engagement Scale was tested using CFA and showed sufficient psychometric properties in our study (CFI = 0.962, TLI = 0.949, RMSEA = 0.081, SRMR = 0.032, Cronbach's alpha = 0.903).

Job performance was measured using a single question: *How would you rate your usual job performance over the past year or two?* (1 = job performance of the worst worker at my job; 10 = performance of the top worker).

Expressive ties were measured by a single statement: *People I work with take a personal interest in me*. Instrumental ties were also measured by a single statement: *My supervisor is helpful in getting the job done*. In both cases, respondents chose one response option on a 4-point Likert scale, where 1 = strongly disagree and 4 = strongly agree.

#### Outcomes—affect-driven behaviors

One outcome related to withdrawal from work was used. Respondents were asked: During the past 30 days, for about how many days did poor physical or poor mental health keep you from doing your usual activities, such as taking care of yourself, work, or leisure? This variable was expressed in days (0–30 days).

To test hypothesis H1.3, we also used a question about experienced difficulties with concentration over the past 30 days while at work (0 = not at all; 7 = frequently). This variable was not, however, used in the modeling of work behaviors as it refers to the experience.

#### Control variables

Studies on employees' reactions to physical characteristics of workspace and perception of privacy showed that two characteristics of employees were of special importance: gender (Newell, 1994) and age (McElroy and Morrow, 2010). Therefore, these two variables were included in the model. Additionally, we controlled for education, race, marital status and location.

### Statistical methods

First, the mean values of the variables of interest were computed for employees working in the open-plan office and those working in different office arrangements. One-tailed two sample mean difference test (without an assumption of equal variances) was executed.

Second, structural equation models to examine how the experience of limited privacy and office density, both associated with working on the open-plan office, relate to employees' reactions. The models were run on a subsample of employees working in the open-plan office (86.8% of the whole sample, see Table 1). Following recommendations by MacKinnon et al. (2002, 2007) and Aguinis et al. (2017), we tested the joint significance of the association between independent and dependent variables (direct effect) and the association between independent variables, mediator, and dependent variable (indirect effect). To this end, structural equation modeling with multiple-item measures (measurement models) for work engagement and personal fit into workspace was used. This approach enabled us to account (at least to some extent) for the measurement error.

To examine whether the scales used in our model were characterized by sufficient measurement properties, CFA (Brown, 2006; Jackson et al., 2009; Kline, 2011) was conducted and Cronbach's alpha coefficient (Bland and Altman, 1997; Tavakol and Dennick, 2011) was computed as a reliability measure. To assess the model fit of CFA, the set of commonly accepted measures including root mean square error of approximation (RMSEA), standardized root mean square residual (SRMR), comparative fit index (CFI), and Tucker-Lewis index (TLI) was used. With respect to RMSEA and SRMR, commonly accepted values should be within the range of 0.00–0.08 (Browne and Cudeck, 1992). With respect to CFI and TLI, it is usually assumed that these statistics should be above 0.9 or 0.95 in order to judge the model as acceptable (Hu and Bentler, 1999).

A series of 18 structural equation models (Bielby and Hauser, 1977; Pearl, 1998; Kline, 2011)—for each of the six outcome variables separately (see section Outcomes—Judgement-Driven Behaviors and Outcomes—Affect-Driven Behaviors) and for each of the three dimensions of physical work environment (visual privacy, acoustical privacy, and office density)—was run. In these models, the same set of mediating and control variables were used; the only difference was in the choice of outcome variables. In terms of testing multiple outcomes, this approach is consistent with an outcomewide regression approach proposed by VanderWeele (2017) to examine and compare an effect of a single exposure on multiple outcomes. Separated models for the three dimensions of the physical work environment were consequential of substantial correlation among them (section Dimensions of Physical Work Environment), which may lead to multicollinearity in the model and negatively affect accuracy of the results.

To examine direct and indirect effects, we applied the effects decomposition (Kline, 2011). Significance of indirect and total effects was tested with bootstrapping method (Aguinis et al., 2017). To examine whether the mediation exists, we adopted the strategy suggested by Aguinis et al. (2017, 676), that is, existence of mediation is supported when the indirect effect is significant, regardless of the presence or absence of a direct effect.

Since our data were of a hierarchical nature [employees clustered in locations; see Table 2 for intraclass correlation coefficient (ICC)], all analyses were conducted adjusting for clustered standard errors.

**Table 2:** Means in the open-plan office and private offices, significance of mean difference test, and intraclass correlation coefficients (ICCs) for study variables.

**Variable**	**Scale**	**Mean**		***p*-value for the one-tailed mean difference test**	**ICC**	**ICC (open-plan office only)**
		**Open-plan office**	**Private office**		
**DIMENSIONS OF PHYSICAL WORK ENVIRONMENT**						
Acoustical privacy	1 = do not interfere at all with my ability to perform my duties and 10 = interfere with my ability to perform my duties to a great extent	5.09	3.81	0.011	0.076	0.070
Visual privacy		4.21	3.48	0.161	0.051	0.035
Office density		3.47	2.48	0.059	0.045	0.049
**AFFECT**						
Irritability	Experienced in the past 30 days while at work: 1 = not at all and 7 = frequently	2.11	1.70	0.064	0.000	0.000
**PERSONAL FIT INTO WORKSPACE**						
Workplace effectiveness	1 = extremely dissatisfied and 10 = extremely satisfied	5.77	7.54	0.000	0.069	0.070
Workplace attractiveness		5.77	7.43	0.000	0.098	0.105
Workplace values		5.96	7.34	0.000	0.121	0.165
Workplace satisfaction		6.16	7.8	0.000	0.093	0.106
**OUTCOMES**						
**Judgement-driven behaviors**				
Work engagement	0 = I do not feel this at all and 6 = I feel this all the time	3.94	4.47	0.000	0.000	0.000
Job satisfaction	0 = not at all satisfied and 10 = extremely satisfied	7.26	7.71	0.043	0.000	0.000
Job performance	1 = job performance of the worst worker at my job and 10 = performance of a top worker	8.36	8.07	0.080	0.014	0.010
Instrumental ties–helpful supervisor	1 = strongly disagree and 4 = strongly agree	3.09	3.24	0.061	0.049	0.049
Expressive ties–people I work with take a personal interest in me	1 = strongly disagree and 4 = strongly agree	2.88	3.1	0.012	0.006	0.000
**Affect-driven behaviors**						
Withdrawal - Number of days with limited ability to do usual activities (e.g. self-care, work, and recreation)	0–30 days	2.11	1.73	0.251	0.001	0.013

Robustness of the results was controlled with respect to the common method bias through the design of the study's procedure (Podsakoff et al., 2003). Although it was not feasible to account for a common rater, a common measurement context, and time biases (as it was of crucial importance to get data from the same persons being in the same measurement context to test the research hypotheses), we proximally and methodologically separated measures of predictors, mediators, and outcome variables. In particular, these groups of measures were located in different sections of the questionnaire and different response scales were used, for example, Likert scales, number of days, intensity scales (see Table 2), with different scale endpoints and different verbal labeling. Additionally, anonymity of respondents and reduction of evaluation apprehension were implemented by adding the sentences in the invitation letter that (i) the choice to participate in this study is completely voluntary; (ii) respondent may choose to not respond to any question(s) without it being held against him/her; (iii) respondent may withdraw without penalty at any time; and (iv) participation will not affect the employment status.

From the analytical perspective, robustness of results was supported by the application of multiple imputations (10 multiple imputations were used) to account for the bias resulting from using the complete case scenario—one of the most commonly used in the literature.

All statistical analyses were performed using Mplus 8.

## Results

Means (in the open-plan office and in private offices), significance of mean difference test, and intraclass correlation coefficients (ICCs) for the study variables are reported in Table 2. They clearly show that employees working in the open-plan office reported significantly more unfavorable working conditions in terms of acoustical privacy (confirming the hypothesis H1.1), workplace effectiveness, attractiveness, and satisfaction (thus confirming the hypothesis H1.4) compared with those working in the private offices. They were also significantly less likely to agree with the statement that the physical space where they worked embodied the values of the organization they worked for (confirming hypothesis H1.4). Additionally, employees from the open-plan office reported significantly lower work engagement and job satisfaction. These results confirmed hypothesis H1.5. Finally, they also reported a lower interest that coworkers took in them (negating the hypothesis H3). The employees working in the open-plan office reported difficulties with concentration, significantly more often than those from private offices (2.57 vs. 1.90, *p*-value = 0.024).

No significant difference between employees from open-plan and private offices was recorded for visual privacy, office density (negating hypothesis H1.1), irritability felt at work (negating hypothesis H1.2), job performance, number of days an employee experienced limited ability to perform usual activities (e.g., self-care, work, and recreation) (negating hypothesis H1.5), and helpfulness of supervisors (negating hypothesis H3).

Generally, hypothesis H1.1 was confirmed in terms of acoustical privacy and not confirmed with regard to visual privacy and office density (however, in the case of office density, *p*-value was only slightly above 0.05, indicating that H1.1 could be confirmed at the significance level of 0.1). Hypothesis H1.2 was negated, though it could be considered marginally significant due to reported *p*-value of 0.064 (if assumed critical significance level was 0.1). Hypotheses H1.3 and H1.4 were confirmed. Hypothesis H1.5 was confirmed with respect to job satisfaction, work engagement, and expressive ties. Additionally, it would be confirmed for job performance at the significance level of 0.1, but indicating better job performance in the open-plan office. H1.5 was definitely negated for affect-driven behavior—limited ability days.

Values of ICC proved that there was a considerable influence of geographic location on reports related to acoustical privacy, workplace effectiveness, attractiveness, and satisfaction as well as values the workspace embodied.

Path estimates for the hypothesized structural models are presented in Table 3 for affect-driven behavior and in Table 4 for judgment-driven behaviors.

**Table 3:** Path estimates of the direct, indirect, and total effects of open-plan office on affect-driven behavior (subsample of open-plan office employees, standardized estimates, and 95% bootstrap confidence intervals).

**Effect**	**Limited ability days**
**ACOUSTICAL PRIVACY**	
**Direct effects**	
Acoustical privacy interfering with task → irritability	0.280^***^
Acoustical privacy interfering with task → affect-driven behavior	0.076
Irritability → affect-driven behavior	0.269^***^
**Indirect effect**	
Acoustical privacy interfering with tasks → irritability → affect-driven behavior	0.075 (0.064; 0.093)
**Total effect**	
Acoustical privacy interfering with tasks → affect-driven behavior	0.152 (0.128; 0.186)
**VISUAL PRIVACY**	
**Direct effects**	
Visual privacy interfering with task → irritability	0.285^***^
Visual privacy interfering with task → affect-driven behavior	0.041
Irritability → affect-driven behavior	0.279^***^
**Indirect effect**	
Visual privacy interfering with tasks → irritability → affect-driven behavior	0.080 (0.065; 0.098)
**Total effect**	
Visual privacy interfering with tasks → affect-driven behavior	0.121 (0.099; 0.015)
**OFFICE DENSITY**	
**Direct effects**	
Office density interfering with task → irritability	0.289^***^
Office density interfering with task → affect-driven behavior	0.016
Irritability → affect-driven behavior	0.287^***^
**Indirect effect**	
Office density interfering with tasks → irritability → affect-driven behavior	0.083 (0.069; 0.105)
**Total effect**	
Office density interfering with tasks → affect-driven behavior	0.099 (0.083; 0.125)

*** *p < 0.001; 95% bootstrap confidence intervals (B = 500 replications)*.

**Table 4:** Path estimates of the direct, indirect, and total effects of open-plan office on judgment-driven behaviors (subsample of open-plan office employees, standardized estimates, and 95% bootstrap confidence intervals).

**Effect**	**Job satisfaction**	**Work engagement**	**Job performance**	**Instrumental ties: *My supervisor is helpful in getting the job done***	**Expressive ties: *People I work with take a personal interest in me***
**ACOUSTICAL PRIVACY**					
**Direct effects**					
Acoustical privacy interfering with tasks → irritability	0.280^***^	0.280^***^	0.280^***^	0.280^***^	0.280^***^
Acoustical privacy interfering with tasks → judgement-driven behavior	0.003	0.046	0.181^**^	0.021	0.021
Irritability → fit into workspace	−0.250^***^	−0.249^***^	−0.244^***^	−0.246^***^	−0.246^***^
Fit into workspace → judgment-driven behavior	0.430^***^	0.357^***^	−0.022	0.328^***^	0.204^**^
**Indirect effect**					
Acoustical privacy interfering with tasks → irritability → fit into workspace → judgment-driven behavior	−0.031	−0.025	0.001	−0.023	−0.014
	(−0.036; −0.024)	(−0.029; −0.023)	(0.001; 0.002)	(−0.025; −0.021)	(−0.016; −0.013)
**Total effect**					
Acoustical privacy interfering with tasks → judgment-driven behavior	−0.028	0.021	0.182	−0.002	0.006
	(−0.032; −0.027)	(0.018; 0.023)	(0.172; 0.201)	(−0.003; −0.021)	(0.006; 0.006)
**VISUAL PRIVACY**					
**Direct effects**					
Visual privacy interfering with tasks → irritability	0.285^***^	0.174^***^	0.285^***^	0.285^***^	0.285^***^
Visual privacy interfering with tasks → judgment-driven behavior	−0.002	0.098	0.116^*^	−0.003	0.067
Irritability → fit into workspace	−0.251^***^	−0.249^***^	−0.244^***^	−0.246^***^	−0.247^***^
Fit into workspace → judgment driven behavior	0.428^***^	0.374^***^	−0.043	0.320^***^	0.221^***^
**Indirect effect**					
Visual privacy interfering with tasks → irritability → fit into workspace → judgment-driven behavior	−0.031	−0.027	0.003	−0.023	−0.016
	(−0.036; −0.028)	(−0.030; −0.025)	(0.003; 0.003)	(−0.025; −0.021)	(−0.017; −0.015)
**Total effect**					
Visual privacy interfering with tasks → judgment-driven behavior	−0.031	0.071	0.119	−0.026	0.051
	(−0.036; −0.028)	(0.066; 0.081)	(0.113; 0.132)	(−0.004; −0.003)	(0.048; 0.057)
**OFFICE DENSITY**					
**Direct effects**					
Office density interfering with tasks → irritability	0.289^***^	0.289^***^	0.289^***^	0.289^***^	0.289^***^
Office density interfering with tasks → judgment-driven behavior	−0.061	0.150^*^	0.189^*^	−0.050	0.081
Irritability → fit into workspace	−0.250^***^	−0.249^***^	−0.244^***^	−0.245^***^	−0.247^***^
Fit into workspace → judgment driven behavior	0.411^***^	0.384^***^	−0.026	0.306^***^	0.221^***^
**Indirect effect**					
Office density interfering with tasks → irritability → fit into workspace → judgment-driven behavior	−0.030	−0.028	0.002	−0.022	−0.016
	(−0.036; −0.027)	(−0.031; −0.025)	(0.002; 0.002)	(−0.025; −0.020)	(−0.018; −0.015)
**Total effect**					
Office density interfering with tasks → judgment-driven behavior	−0.091	0.122	0.191	−0.072	0.065
	(−0.110; −0.081)	(0.112; 0.137)	(0.174; 0.213)	(−0.081; −0.067)	(0.060; 0.075)

*** *p < 0.001*,

** *p < 0.01*,

* *p < 0.05; 95% bootstrap confidence intervals (B = 500 replications)*.

In the case of affect-driven behavior, that is, limited ability days, the paths from each examined dimension of open-plan office and irritability were significant (beta_acousticalprivacy_ = 0.280, beta_visualprivacy_ = 0.285, and beta_officedensity_ = 0.289, *p* < 0.001), indicating moderate, but significant detrimental effects associated with increased irritability and confirming hypothesis H4. Similarly, paths from irritability to limited ability days were also significant for all examined dimensions of workspace (beta_acousticalprivacy_ = 0.269, beta_visualprivacy_ = 0.279, and beta_officedensity_ = 0.287, *p* < 0.001), indicating a negative correlation between irritability and number of days with limited ability to perform usual activities (also work). This finding confirmed hypothesis H6. Contrary to what was hypothesized in H8 regarding a direct effect, no direct effect of acoustical privacy, visual privacy, or office density was found significant. Nonetheless, both indirect effects—through irritability—and total effects were found significant for each of the analyzed effects representing dimensions of open-plan office (none of the 95% bootstrap confidence intervals included 0). It implied that negative perception of acoustical privacy, visual privacy, or office density is associated with higher number of limited ability days, thus confirming hypothesis H8 with respect to an indirect effect.

The paths from negative perception of the dimension and irritability to judgement-driven behaviors were significant for each dimension of the open-plan office and each judgement-driven behavior. This supported the link between detrimental effects of open-plan office in terms of either acoustical privacy, visual privacy, or office density and irritability. This finding confirmed hypothesis H4.

Similarly, significant negative association between irritability and perception of fit into workspace was found for each of 18 analyzed models. This confirmed hypothesis H5 about detrimental effect of irritability on perception of fit into workspace. With regards to perception of fit into workspace and its correlation with judgement-driven behaviors, positive correlation was found for job satisfaction, work engagement, and expressive and instrumental ties, regardless of the type of effect (i.e., visual privacy, acoustical privacy, and office density). No effect was found for job performance. It implied that hypothesis H2 was confirmed for four out of five behaviors examined.

No direct effect of privacy—either acoustical or visual—was found on job satisfaction, work engagement, and expressive and instrumental ties. Direct effect was, however, found for job performance. In this case, a significant positive correlation was found that implied a positive effect of limited privacy (both acoustically and visually) on own assessment of job performance. Similar effect was found for office density perceived as interfering with performing tasks yielding increased job performance and higher work engagement. In all above cases, hypothesis H7 was negated due to the direction of the association.

All indirect effects were found to be significant and indicated possible negative impact of limited privacy and increased office density on employees' behaviors. This confirmed hypothesis H7 with regard to indirect effects of limited privacy and increased office density for job satisfaction and work engagement and negated hypothesis H3 for instrumental and expressive ties. Hypothesis H7 was not confirmed for job performance, for which the effects were positive. Similarly, total effects were found significant for each examined behavior. However, they pointed out to negative effects of acoustical privacy, visual privacy, and office density on job satisfaction and instrumental ties expressed by the fact that supervisors were helpful in getting the job done. With respect to other behaviors—work engagement, job performance, and expressive ties, that is, coworkers expressing personal interests in themselves, the total effects were positive. It implied that hypothesis H3 was confirmed for expressive ties, suggesting that working in an open-plan office generally favors personal (nonwork-related) relationships among coworkers, though the effect is not direct. This hypothesis was negated for instrumental ties.

In all models for judgment-driven behaviors—excluding model for job performance—significant positive correlations between perception of fit into workspace and respective behaviors were found. This finding corroborated hypothesis H2 about the positive role of workspace attractiveness for performance outcomes.

## Discussion

Survey on employees of a top architectural company was instrumental for the assessment of the impact that an open-plan office and its three characteristics (acoustical privacy, visual privacy, and office density) may have on mood at work, perception of fit into workspace, job satisfaction, work engagement, job performance, social relations at work, and withdrawal at work (self-reported limited ability days). Following H1, our data pointed to significant differences between employees working in an open-plan office and those working in private offices. The private office employees excelled in terms of perception of privacy, office density, and fit into workspace. Open-plan office employees more frequently reported irritability, problems with concentration, and worse performance outcomes, but not in terms of withdrawal at work due to health-related limitations.

Our findings corroborate conclusions of De Croon et al. (2005) that workplace openness is negatively related to perceptions of visual and acoustical privacy. Further, if these perceptions are negative, that is, while working in open-plan offices impacts possibilities of concentrating, interferes with performing duties, and decreases satisfaction with workplace, the effects are detrimental to performance outcomes as noted by De Croon et al. (2005), Veitch et al. (2007), and Sundstrom et al. (1980), among others.

Following the affective events model of employee behavior in an open-plan office, by Ashkanasy et al. (2014), supplemented by elements of the spatial model of work relationships by Khazanchi et al. (2018), we hypothesized that employee behaviors (judgment-driven and affect-driven, such as withdrawal at work) and work relationships in the open-plan office may be mediated by negative emotions (such as irritability) and perception of fit into workspace (only in the case of judgment-driven behaviors). Our study showed that direct effect of each of three examined features of the open-plan office (acoustical privacy, visual privacy, and office density) was found insignificant (the only exception was job performance for which a positive direct effect was found). However, indirect effects for all analyzed behaviors were significantly negative (with exception of job performance where the positive effect was recorded). This indicated that in the investigated population, perception of privacy and office density was an important factor usually influencing work-related behaviors and withdrawal at work adversely, thus confirming hypotheses H7 and H8. In particular, if working in an open-plan office affected experience of privacy and induced perception of crowded office space, due to increased irritability (H4) and reduced perception of fit into workspace (H5), it negatively affected all analyzed outcomes (but job performance).

We also showed that employees' perception of lack of privacy at work and excessive office density, resulting from the lack of architectural privacy related to working in the open-plan office, may negatively affect their formal work relationships with supervisors and positively affect personal work relations among coworkers. In particular, our study showed that the impact on social relationships is not direct (thus negating hypotheses H3 and H8 with respect to direct effect). It is mediated by irritability, which yields negatively to work behaviors and by fit into workspace, which if satisfied, yields positively. We showed that while indirect effect of each of the open-plan office dimensions on social relationships is negative, total effect of each dimension on informal relationships among coworkers (i.e., expressive ties) could be positive. Total effect on professional relationships (i.e., internal ties) was found to be negative.

Since no significant direct effects of privacy and office density experiences were found on analyzed behaviors (with the exception of job performance) and withdrawal at work, a (full) mediation role of irritability and fit into workspace (only for judgment-driven behaviors) was confirmed. In this regard, our analysis expands the current state of knowledge by showing that the effect of working in the open-plan office is not only direct, but also mediated by emotions and fit into workspace at workplace. In this respect, our study is an empirical validation of Ashkanasy et al.'s (2014) model. It also corroborates Veitch's (2018) findings that positive opinions about attractiveness of workspace translate into favorable work performance, which confirmed our hypothesis H2.

Similarly to Seddigh et al. (2014), who reported no significant differences in general health of employees between different types of open-plan offices, and contrary to Bodin Danielsson et al. (2014), who reported a significant higher risk for short-term sickness among employees working in open-plan offices compared with other employees, our results indicated no significant differences between open-plan and private offices in terms of health (the number of limited ability days due to health problems). No direct impact of any of the dimensions of open-plan office (acoustical privacy, visual privacy, and office density) was found either. Nevertheless, by reporting significant negative indirect effects, we provided more details on the mechanism through which the risk of ill health arises—negative emotions and decreased perception of fit into workspace.

In the analyzed population, in which 86.7% of employees declared having a job that requires creativity, little evidence was found to support supposition that an open-plan office plan can provide additional advantage in the knowledge-creation process for architects and designers in the organization analyzed. Instead, our results are rather in line with those of Kim and de Dear (2013) and Samani et al. (2017). They found that although open-plan offices may contribute to interactions and teamwork, lack of visual and acoustical privacy, consequential to distractions resulting from crowding, will rather negatively influence work-related behaviors. Additionally, work in an architecture company could be perceived as complex and thus our results support findings of Block and Stokes (1989) suggesting that employees involved in performing complex tasks are more satisfied and efficient in private offices than in open-plan offices.

### Strengths and limitations

Mixed findings of the literature have been usually attributed to different work requirements of specific jobs (Pejtersen et al., 2006). Employees whose job requires high level of concentration have reported more distraction in all types of offices, but cell offices (Seddigh et al., 2014). Jobs perceived as depending on team work and knowledge sharing are indicated as those whose performance should not be affected by distracting effect of lack of privacy related to open-plan office (Pejtersen et al., 2006). One possible example of such a job—architects—was proposed, though not tested, by Pejtersen et al. (2006). Our study provides a unique opportunity to examine this assertion.

As for our other contributions to the literature, by examining the impact of working in the open-plan office in 20 locations of the same architectural company, this study benefits from examining an employee pool with similar job requirements defined by the nature of the business, but based in different local offices. Additionally, as architects, supported by ergonomists, interior designers, and behavioral scientists, are mainly responsible for shaping the modern office space (Knight and Haslam, 2010; Vartanian et al., 2013), this study provides an opportunity to examine functionality and impact of a particular architectural solution, that is, open-plan office, on performance outcomes, health, and social relationships among its designers. Our results suggest that the impact is rather adverse and induces negative emotions—such as irritability—and negative attitudes—such as perception of diminished workspace fit, which, in turn, affect the final behavioral outcomes.

The positive total effect of acoustical privacy, visual privacy, and office density on expressive ties associated with private relationships or friendship was found in our study. Although this finding corroborates relationship-building effects of working in open-plan office formulated by Khazanchi et al. (2018), it also indicates that future studies should focus on explaining the mechanisms through which a negative emotion, such as irritability (but also anger and frustration) can be regulated or compensated to offset negative impact of workspace characteristics.

Application of irritability in our study, instead of anger and frustration, originally proposed by Ashkanasy et al. (2014), is another contribution of this paper. We argue that in studies where effects of working in open-plan office and thus of rather incessant experience of low privacy and high office density are of interest, an application of a prolonged emotional state reflecting reactions to changes in the environment—such as irritability (Kuczynski and Kolakowsky-Hayner, 2011)—is more accurate to capture examined effects of the work environment.

Finally, to our knowledge, this is the first paper to provide an empirical validation of Khazanchi et al.'s (2018) idea of relationship-building and relationships-straining effects of workspace design with distinction between expressive and instrumental ties. Although Khazanchi et al. (2018) did not include emotions into relationships-straining, we believe that the negative emotion of irritability applied in this article can be perceived as another relationship-straining mediator. Thus, the negative impact of open-plan office expressed by lack of privacy and excessive office density on work-related relationships (instrumental ties) at work, corroborated by our results, is in line with the model of spatial model of work relationships with respect to relationship-straining effects. Our findings are in line with Oldham and Brass (1979) and Samani et al. (2017) that employees working in an open-plan office find it difficult to engage in private conversations with supervisors and supervisors reported difficulties with providing evaluative feedback.

Despite these strengths, our study has some limitations. First, our use of cross-sectional data restrains us from drawing causal inferences. Second, an application of several instead of one structural equation model may be perceived as a caveat. Although our approach was consequential of both multicollinearity issues (detected between dimensions of physical work environment and between performance outcomes) and our desire to disentangle the effects of different dimensions the physical work environment may have, an application of a summary metric of physical workspace characteristics and human performance should be considered in future studies. Our attempts in this respect, however, did not provide satisfactory results in terms of fit to the theoretical assumptions of the structural equation modeling and the conceptual model tested. Third, as indicated by Bodin Danielsson et al. (2014), the impact of the open-plan office on sick days can differ between different types of open-plan offices. We were not able to examine the influence of different types of open-plan offices on experience of privacy. However, findings of Seddigh et al. (2014) suggest that differences in health and performance outcomes among types of open-plan offices may not be significant. Fourth, we were not able to control for open-plan office characteristics, such as physical measurements of proximity and noise level, as they were not collected. We collected only perceptions of privacy and office density. Finally, in our study, significantly positive direct and indirect effects of experience of lack of privacy and office density on job performance were found. This interesting finding might have resulted from the excessively overly positive assessment of own job performance, called self-assessment bias, which has been known to be present in a wide variety of work situations (Walfish et al., 2012). Unfortunately, we were not able to control for it. Nevertheless, future studies should consider accounting for this effect.

Future studies should also look into the validation of the positive effect of open-plan office on job performance. As we formulated our questions about the open-plan office in a negative way (i.e., how much each of the physical characteristics of workspace interfered with the ability to perform duties), it influenced us to expect negative effects. It might be, however, the case that with the positively phrased questions about workspace design, the positive effect can be substantiated.

### Conclusions

Open-plan office was developed from a perspective of cost accounting for the environmental impact, better space efficiencies for sustainability, and the desire of companies to encourage social collisions and collaborative teams to accelerate innovation and enhance job performance. The impact of this design on humans is not only less certain in terms of the costs related to the absenteeism, presenteeism, attraction, and retention of talent, but also health and productivity. We join De Croon et al. (2005) and Jahncke et al. (2011) and argue that newer office designs should offer a mix of open-plan office—to meet needs for interactions, communications, teamwork, and knowledge sharing—and private spaces to protect against acoustical distractions, visual distractions, and crowding, and to enable the still-needed individual, headsdown work.

## Author contributions

DW-B performed data analysis and interpreted the result, drafted the manuscript, and approved the final version of the manuscript. ZD performed data analysis, contributed to interpretation of the results, revised the manuscript, and approved the final version of the manuscript. EM developed the study design, contributed to interpretation of the results, revised the manuscript, and approved the final version of the manuscript.

### Conflict of interest statement

The authors declare that the research was conducted in the absence of any commercial or financial relationships that could be construed as a potential conflict of interest.
